# How health motivation moderates the effect of intention and usage of wearable medical devices? An empirical study in Malaysia

**DOI:** 10.3389/fpubh.2022.931557

**Published:** 2022-08-15

**Authors:** Naeem Hayat, Noor Raihani Zainol, Anas A. Salameh, Abdullah Al Mamun, Qing Yang, Mohd Fairuz Md Salleh

**Affiliations:** ^1^Global Entrepreneurship Research and Innovation Centre, Universiti Malaysia Kelantan, Kota Bharu, Malaysia; ^2^Faculty of Entrepreneurship and Business, Universiti Malaysia Kelantan, Kota Bharu, Malaysia; ^3^College of Business Administration, Prince Sattam Bin Abdulaziz University, Al-Kharj, Saudi Arabia; ^4^UKM - Graduate School of Business, Universiti Kebangsaan Malaysia, Bangi, Malaysia

**Keywords:** wearable medical device, intention, adoption, value, health motivation, product value, Malaysia

## Abstract

Mobile technology is popular because it facilitates users in multiple ways. This technology is revolutionising the healthcare industry, and mobile-based wearable medical devices (WMDs) are becoming common. Technology adoption is always challenging, and technology value is based on the technology attributes and personal inclination towards using the technology. This study investigates how the perceived product value is influenced by compatibility, cost, personal privacy, technology accuracy, and usefulness. The perceived product value instigates the intention to use wearable medical devices and health motivation (HMT), and the intention to use promotes the adoption of WMDs. Furthermore, the relationship between the intention to use and the adoption of WMDs is moderated by HMT. The current work employed a cross-sectional research design, and data were collected through an online survey of Malaysian adults. Data analysis was performed using partial least squares structural equation modelling (PLS-SEM). The analysis revealed that the WMDs' compatibility, cost, and technology accuracy significantly influenced the WMDs' value. Besides, the perceived product value impacted the intention to use WMDs, while HMT and intention to use WMDs insignificantly influenced the adoption of WMDs. Finally, HMT significantly moderated the relationship between the intention to use and the adoption of WMDs. This study also reports the limitations and future research opportunities.

## Introduction

Technologies are becoming a crucial part of everyday life, and health-related technologies have become popular with the advent of COVID-19. The popularity of mobile devices has led to smart wearable devices whereby people can effortlessly use their devices anytime and anywhere (1). Dehghani et al. (2) have postulated that about 500 smart devices are available for entertainment, medical, fitness, gaming, industrial, and lifestyle purposes. Smart glasses, clothing, smart watches, jewellery, headband, and wristbands are a few examples of smart devices (3).

The diffusion of mobile devices is evident since ~70% of the world's population use mobile devices (1). Currently, mobile devices are equipped with sensors, actuators, global positioning systems, and accelerometers that empower users to monitor their health conditions in real-time (4). The combination of biosensors and computing technologies offers a portable, non-invasive and unobtrusive monitoring of a user's physiological data to determine his or her health condition (5).

With the development of artificial intelligence, big data and other technologies, wearable medical devices have broken through the limitations of the use of many traditional medical devices and brought new innovative directions to the medical device industry and disease prevention and control methods. Meanwhile, the technology development and promotion of wearable medical devices have become a growing business element for technology companies (6). Wearable medical devices have become a growing business aspect for technology firms. The market value for wearable medical devices s will touch the USD 30 billion mark by the end of 2022 (7). Health-based WMDs facilitate the users to check their health instantly, physical activities, food intake, nutrient value, sleeping cycles, and heartbeat rate, along with their general health condition (8). A variety of wearable medical devices are available in the market in the form of smartwatches, smart bands, and phone-based tools (1). Global consumers enjoy the services of wearable medical devices such as Fitbit, Apple Watch, Honor Smart Watch, and Samsung Galaxy Watch (9). These smart wearable medical devices empower users to manage their personal health at the convenience of their homes.

As an emerging healthcare delivery and technology economy, Malaysia needs a viable means to deliver quality healthcare services. The 12th Malaysia Plan aims to digitally transform the national healthcare landscape and delivery (10). Moreover, the rising healthcare cost puts pressure on individuals, and people are looking for alternatives to manage their personal health in a quality, convenient, and affordable manner. Malaysian users are experiencing a 13.5% inflation in medical services because the number of qualified doctors and nurses for 1,000 individuals is 1.5 and 3.5, respectively (11). Thus, wearable medical devices (WMDs) empower users to achieve wellness and deliver convenience in managing personal health.

Teahcnology attributes always builds the necessary value for the prospective users that instigates the use behaviours. However, for healthcare technologies the personal health motivation facilitates the use of medical technology and offer the better health quality among the people having higher health motivation (12). Health motivation drives the consumer behaviour and can facilitates the adoption. The current study aims to explore the emergence of perceived product value (PPV) influenced by certain factors (compatibility, cost, personal privacy, accuracy, and usefulness). The perceived product value prompts the intention to use WMDs, while the intention to use and health motivation (HMT) influence the adoption of WMDs. Besides, the relationship between the intention to use and the adoption of WMDs is moderated by HMT.

## Literature review

### Theoretical foundation

Numerous theories have been utilised to explain or predict health-related behaviour predicated on the belief that social, technological, behavioural, or psychological factors impact health (1, 4). The Technology Acceptance Model (TAM) is a well-known theory for assessing users' acceptance of new technology (13). According to this theory, two main factors influence a person's decision to adopt innovative technology, i.e., perceived ease of use and perceived usefulness (6). Despite significant criticism, TAM is widely regarded as one of the most suitable theoretical frameworks for describing consumers' intention to use digital technologies (14). Consumers are more likely to utilise a technological product if they consider it useful, beneficial, helpful, and simple to use (1), which then influences the intention to adopt the technology (9). The technology attributes compatibility, accuracy, and usefulness instigate the intention to use a technology (12). Users' intention to use and the adoption of WMDs have been studied using TAM (7).

According to Bandara and Amarasena (15), the perception of technology value builds on the technology attributes. The perceived value theory instigates that the consumer-level perceived technology value is based on assessing the market value of the product utility minus the cost associated with the technology (16). Besides, the technology value may include the technology users' functional, social, and emotional values (17). Technology attributes of compatibility, usefulness, and accuracy nurture consumers' positive perception and perceived value (18). Nevertheless, specific technology attributes may lead to a negative perception and build an unfavourable value towards using the technology (19). For example, cost and personal privacy issues can negatively influence the technology's value. Similarly, price and time can instigate a low perception of technology value and curtail the use of the technology (20).

The intention to use and the adoption of WMDs are substantially associated with the perception of healthcare product value (2). When people believe they are at risk of acquiring a health problem, they tend to purchase healthcare technology services to protect themselves from health risks (17). Individuals are influenced into becoming potential users of WMDs by the technological aspects of the products. A WMD's technological aspects, including functionality, ease of use, accuracy, compatibility, and convenience, can influence the user's actual behaviour (1). Numerous studies have explored and confirmed that intention leads to users' acceptance of wearables, besides highly suggesting the development of further research with more antecedents (3, 7).

### Hypotheses development

#### Perceived compatibility and perceived product value

Perceived compatibility (PCM) is defined as the extent to which the functionality of a WMD is compatible with the product's function, users' lifestyles, and current needs (12, 21). Moreover, compatibility relates to how well users' existing values, attitudes, beliefs, behaviours, and current and historical experiences are aligned with their usage of a WMD (22). Product value, as a form of perception, represents the consumer's perception of a product's money and functionality and thus influences the consumer's intention for a new product or technology (23). Compatibility has been recognised as an essential variable in adopting new technologies in previous studies (21, 22). It is a crucial factor in the success of WMD adoption, as WMD services must conform to and be compatible with users' expectations and lifestyles to be embraced successfully (12). When a WMD is more compatible with users' experiences and lifestyles, it will be easier for them to understand how to use it and create subjective value for technology products (24). Thus, compatibility can affect product value since the more compatible a device is, the higher the perceived value it may carry. As such, the following hypothesis is proposed:

*H1a: PCM positively influences the PPV of WMDs*.

#### Perceived cost and perceived product value

Perceived cost (PCT) is a critical aspect that affects whether people will use smart devices (9). PCT refers to a person's belief that using WMDs would cost money (25). People will be reluctant to use WMDs if the devices are believed to be expensive. Even though WMDs assist and benefit users by capturing and reporting health data that may be tracked, the devices are too expensive for some users (19). In fact, high pricing is a barrier for users and will negatively influence device acceptability (9). Nonetheless, if users believe that the advantages of using WMDs outweigh the cost, their perceived value rises, motivating users to adopt WMDs (7). Users tend to seek high-quality products at a decent cost (20). The acceptability of a product's value is often determined by its price or cost. Thus, the following hypothesis is formulated:

*H1b: PCT positively influences the PPV of WMDs*.

#### Perceived personal privacy and perceived product value

Perceived personal privacy (PPP) is a crucial concern for healthcare wearable technology adoption (19) because users must register sensitive data concerning their health status to get valuable services (14). Therefore, users need to secure their personal information from inappropriate outflows. PPP refers to the extent to which people consider their personal information will not be misused or distributed to others without their consent (26). Privacy in mobile healthcare is characterised as the ability to govern the acquisition and dissemination of personally identifiable health data (3). Information about users' health conditions is captured and maintained on a database when they utilise WMDs, which raises privacy issues among consumers (26). Kim and Ho (18) postulate that healthcare technology privacy is directly associated with utilising wearable healthcare technology. Nevertheless, Sergueeva et al. (7) indicate that it does not affect wearable technology devices. As such, it is vital to explore the impact of PPP on the product value of WMDs. Based on the discussed literature, the following hypothesis is proposed:

*H1c: PPP positively influences the PPV of WMDs*.

#### Perceived technology accuracy and perceived product value

Technology accuracy is one of the major factors that influence the PPV of WMDs (15). The degree to which customers think the information given by the wearable healthcare technology about their health status is accurate and credible is called health information accuracy (26). Technology accuracy refers to the precision and reliability of information (27). For the users, raising the accuracy of the product value is critical. People will be influenced to become potential users of WMDs by the technological aspects of the products and their effects on society (14). Users' propensity to engage in health-related informed decisions acquired from WMDs is positively influenced by the accuracy of the health information offered by the wearable health technology devices (26). WMD technology accuracy can influence product value. Hence, the following hypothesis is put forth:

*H1d: PTA positively influences the PPV of WMDs*.

#### Perceived usefulness and perceived product value

Perceived usefulness (PUF) concerns an individual's belief that utilising a certain system will improve his or her work performance (13) and is deemed one of the most vital factors that influence users' desire to use digital technologies (14). PUF is defined as the user believing that utilising a wearable device would be advantageous to his or her health (12, 22). The degree to which users consider a WMD simple to use would impact their PPV and intention to adopt it. WMDs that are valuable and straightforward to use are more likely to be adopted by consumers (5). Furthermore, when users or consumers perceive that WMDs can help them improve their health, their positive expectation boosts their desire to utilise them (14). Users are more willing to use a wearable gadget if they believe it will allow them to conveniently check their health condition and efficiently nurture a healthy lifestyle (6). Hence, the following hypothesis is proposed:

*H1e: PUF positively influences the PPV of WMDs*.

#### Perceived product value and intention to use WMDs

Perceived value is a crucial component in intended behaviour and has been recognised as an essential marketing winning strategy in recent years (16, 28). Prior studies define perceived value as a user's entire evaluation of a product's utility based on perceptions of what is received and what is given (24, 29). In other words, perceived value involves what is received in terms of benefit, quality, and utility and what is given in terms of price and costs (28). The perceived value of wearable devices is described as a potential customer's general perception of wearable devices that is focused on their benefits and costs (22). Since this perception is a part of customers' behaviour, empirical investigations have demonstrated that perceived value could effectively elucidate behavioural decisions (16). If a technology produced or exceeded the desired health improvement or service expectation, the user would be satisfied; however, the user would be unsatisfied if the outcomes were unexpected (22). Users and marketers have often emphasised the significance of customer perceived value because it is one of the most significant drivers of behaviour intention (5). Therefore, the current study formulates the following hypothesis:

*H*_2_*: PPV positively influences the intention to use WMDs*.

#### Health motivation and intention to use WMDs

HMT is defined as “a burning desire to engage in preventive health activity” (2). Individuals who are motivated about their health are more likely to utilise and embrace WMDs (12). Prospective users might also be more likely to purchase healthcare products. Since WMDs can be used to sustain and enhance health, users who are more motivated and engaged in healthcare are more inclined to have a greater intention to adopt WMDs (1). When users have a higher interest in health and are very motivated to research ways to enhance their health, they are more likely to learn about the benefits of wearable healthcare technology, increasing the likelihood of adoption (26). Thus, the following hypothesis is formulated:

*H*_3_*: HMT positively influences the use of WMDs*.

#### Intention to use WMDs and usage of wearable medical device

Intention to use is the degree to which an individual has developed conscious plans to conduct or not a specified future behaviour (2). Besides, intention to use is regarded as a consumer's desire to use innovative technology products such as WMDs and is determined by factors that influence actual behaviour (1). The willingness to use health-related technology is a strong predictor of actual adoption. Meanwhile, the intention to use health-based personal devices predicts WMD adoption (30). It is deemed the best predictor of adopting health-based wearable devices (17). A consumer or user is more likely to adopt innovative technological healthcare devices when the behavioural intention is high. Hence, this study proposes the following hypothesis:

*H4: Intention to use WMDs positively influences the use of WMDs*.

#### Moderation of HMT

Motivation significantly influences how people feel and what they want to do. People are more prone to engage in behaviours that prioritise their objectives while avoiding activities that could lead to disaster (31). HMT is a desire to change one's health and quality of life by implementing a healthy lifestyle (32). Changing lifestyle entails both awareness and worry about one's health, which can be facilitated by WMDs (2). Users who are motivated about their health are more likely to utilise and embrace WMDs (12). Health-conscious individuals believe they are more likely to engage in preventative behaviour and manage their health conditions daily (18). When people are more motivated to improve their health, they are more inclined to use devices to monitor their health (7). The effect of HMT on WMD adoption has received little attention in past research. Thus, the current work investigates the moderating role of HMT in the association between the intention to use and the adoption of WMDs to validate the direct positive effect of HMT on WMD usage. The following is formulated:

*HM1: The relationship between intention and the adoption of WMDs is positively moderated by HMT*.

## Research methodology

### Sample size calculation and data collection

A self-administered questionnaire was used in this study, and an online questionnaire link was posted through Google form for data collection. The questionnaire contained a background introduction to the study, keyword parsing, background questions, and measurement questions. The current work employed a cross-sectional research design, and data were collected through an online survey of Malaysian adults. In addition to the informed consent, wearable medical devices were explained in the introduction to this questionnaire to help respondents understand the survey content of the questionnaire. All respondents were informed of their right to discontinue their responses at any time before they formally participated. All incomplete responses were eventually marked as invalid. The corresponding number of valid responses was 1,160, which far exceeded the minimum sample size calculated using G^*^Power 3.1 with a power of 0.95, α of 0.05 and an effect size of 0.15. Combining the eight predictors of the study, the minimum sample size was calculated to be 160 (33). The data collection was performed online between January 2022 to March 2022 from the Malaysian Adults.

### Measurement scales

This study's measurement scales were derived from recognised and valid scales. Four items taken from Li et al. (32) were used to assess HMT. A sample question item was “I have good knowledge to prevent health issues.” On the other hand, PCM was estimated using four items from Yang et al. (6), and a sample question item was “I think using wearable medical devices suits my way of managing health at home.” Four items were utilised to gauge PCT for the WMDs (34), and a sample question item was “I am pleased with the wearable medical devices prices.” Next, PPP was evaluated using four items from Gao et al. (19). A sample question statement was, “It would be risky to disclose my personal health information to vendors providing medical wearable devices.” Perception of technology accuracy was measured using four items from Alam et al. (17), and a sample question item was “I feel confident that wearable medical devices are offering error-free results.”

Meanwhile, PUF for the WMDs was estimated using four items extracted from Yang et al. (6). A sample question statement was “Using the wearable medical device is beneficial to manage health.” On the other hand, the perceived product value was evaluated using four items taken from Kim et al. (34), and a sample question item was “I think using the wearable medical device is worthwhile.” For the intention to use WMDs, four items from Alam et al. (17) and Gao et al. (19) were employed to gauge it. A sample question was “I would be willing to develop the habit of using wearable medical devices.” Finally, the use of WMDs was assessed using a single-question item. All the questionnaire items relating to exogenous variables were marked using a five-point Likert scale, whereas endogenous variables were graded based on a seven-point Likert scale. In the research design stage, using distinct Likert scales for input and outcome variables solves the issue of common method variance (CMV) (35).

### Common method variance

Based on Podsakoff et al. (35), Harman's single factor test was conducted. The single factor accounted for 30.9%, i.e., below the recommended threshold of 40.0%, suggesting that CMV was not an issue in the current study (35). Furthermore, CMV was evaluated in the current study *via* the full collinearity test (36). All the study constructs regressed on the common variable. The variance inflation factor (VIF) values for PCM (1.891), PCT (2.321), PPP (1.633), PTA (2.287), PUF (2.349), PPV (3.280), HMT (2.985), intention to use WMDs (2.842), and use behaviour (2.101) were below 3.3, confirming the absence of bias from the single-source data (36).

### Multivariate normality

Multivariate normality for the current study was evaluated using the Web Power online tool (source: https://webpower.psychstat.org/wiki/tools/index). The premeditated Mardia's multivariate skewness and kurtosis coefficient and *p*-values demonstrated that the study data had a non-normality issue since the *p*-values were below 0.05 (37).

### Data analysis method

The descriptive analysis was performed with the SPSS 23 software, and with multivariate non-normality issue in the dataset, the study utilised PLS-SEM. Hair et al. (38) have suggested that variance-based structural equation modelling (SEM) is adopted to analyse the causal-predictive, explanatory nature, and non-normality issues to explain the variance in the structural equation model's dependent constructs in-depth.

The Smart-PLS 3.2 program was employed to analyse the data collected. PLS-SEM is a multivariate exploratory method for analysing integrated latent constructs' path structure (38). It allows researchers to work well with the non-normal dataset with small data points. Furthermore, PLS-SEM is a casual-predictive analytical tool to execute complex models with composites and no specific assumption of goodness-of-fit static requirements (39). In this study, the PLS-SEM analysis was performed in two phases. The first stage dealt with model estimation, where the model's construct reliability and validity were evaluated (38). Meanwhile, stage two evaluated the path values and model fitness statistics in which the *r*^2^, *Q*^2^, and effect size *f*^2^ explained the endogenous construct's change caused by the exogenous constructs (38).

## Findings

### Demographic profile of respondents

The descriptive analysis was performed, and the results are presented in Table 1. Most of the respondents were females (52.1%). The respondents' age ranges were as follows: 20–30 years (6.8%), 31–40 years (8.9%), 41–50 years (47.4%), 51–60 years (30.4%), and 60 years old above (6.5%). In terms of education, most respondents possessed a bachelor's degree (36.9%), followed by a diploma (23.4%), secondary school certificate (17.0%), master's degree (16.6%), and doctoral degree (6.0%). Next, for the respondents' average monthly income, 12.5% had an average monthly income of below US$ 250, 22.8% had an average monthly income between US$ 250–450, and another 21% had an average monthly income between US$450–650. Meanwhile, 23.2% had an average monthly income between US$ 651–900, 9.7% had a monthly income between US$ 901–1,200, and 10.8% had a monthly income of above US$ 1,200. To further help respondents understand the research context of this study and determine the experience of the study's population with medical devices. This study investigated respondents' experiences using Medical Devices in the background questions. The results showed that 24.1% of the respondents used medical devices for more than half a year, 17.2% used them for more than 1 year, 7.8% for more than 3 years, 6.7% for more than 5 years, while 23.4% never used any medical device. The respondents resided in Sarawak (13.4%), Pulau Pinang (11.0%), Kuala Lumpur (10.7%), Kedah (9.9%), Kelantan (9.8%), Johor Bahru (9.4%), Pahang (9.2%), Terengganu (9.2%), and others (17.3%).

**Table 1:** Demographic characteristics.

	***N***	**%**
**Gender**		
Male	556	47.9
Female	604	52.1
Total	1,160	100
**Age**		
20–30 years	79	6.8
31–40 years	103	8.9
41–50 years	550	47.4
51–60 years	353	30.4
Above 60 years	75	6.5
Total	1,160	100
**Living province**		
Kuala Lumpur	125	10.7
Pahang	107	9.2
Penang	128	11.0
Sarawak	155	13.4
Teregganu	105	9.1
Johor Bahru	110	9.4
Kelatan	114	9.8
Kedah	115	9.9
Others	201	17.3
Total	1,160	100
**Education**		
Secondary school certificate	197	17.0
Diploma	272	23.4
Bachelor's degree or equivalent	428	36.9
Master's degree	193	16.6
Doctoral degree	70	6.0
Total	1,160	100
**Average monthly income**		
Below US$ 250	145	12.5
US$ 250–US$ 450	265	22.8
US$ 450–US$ 650	244	21.0
US$ 651–US$ 900	268	23.2
US$ 901–US$ 1,200	112	9.7
More than US$ 1,200	126	10.8
Total	1,160	100
**Used medical device**		
Never used	272	23.4
More than 1 month	241	2.8
More than half a year	279	24.1
More than 1 year	199	17.2
More than 3 years	91	7.8
More than 5 years	78	6.7
Total	1,160	100

***Source:** Author's data analysis*.

*1USD = 4.2 Malaysian RM*.

### PLS-SEM analysis and results

#### Reliability and validity

In the first stage of the PLS-SEM analysis, all the constructs' reliability and validity were evaluated (presented in Table 2). Reliability was assessed using Cronbach's alpha (CA). CA values above 0.70 are considered acceptable and suitable for predicting appropriate reliability (39). In this study, the minimum score was achieved by PPV with a score of 0.781. Dijkstra-Hensele's *rho* (rho_A) was the second reliability measure utilised to evaluate the reliability of the constructs. A DH rho score above 0.70 is deemed satisfactory (39). PCN achieved the minimum DH rho score, i.e., 0.855. Meanwhile, the composite reliability (CR) score needs to be above 0.70 to confirm a construct's reliability and be viewed as acceptable (39). The minimum score was attained by PCT (0.867). Next, the average variance extracted (AVE) for constructs needs to be above 0.50, whereby values above 0.50 confirm good convergent validity (38). Furthermore, the VIF value must be <5.5 (40). In this study, no issue of multicollinearity was observed as the VIF values were <3.3 (36).

**Table 2:** Reliability and validity.

**Variables**	**No. of Items**	**CA**	**rho_A**	**CR**	**AVE**	**VIF**
PCM	3	0.891	0.855	0.916	0.785	3.136
PCT	3	0.795	0.929	0.867	0.690	1.674
PPP	3	0.975	0.920	0.983	0.951	2.868
PTA	4	0.962	0.949	0.971	0.690	1.980
PUF	5	0.964	0.900	0.957	0.882	2.603
PPV	3	0.781	0.846	0.869	0.690	1.000
HMT	3	0.918	0.916	0.936	0.830	1.084
IWD	3	0.964	0.969	0.977	0.933	1.290
UWD	1	1.000	1.000	1.000	1.000	-

*PCM, Perceived compatibility; PCT, Perceived cost; PPP, Perceived privacy protection; PTA, Perceived technology accuracy; PUF, Perceived usefulness; PPV, Perceived product value; HMT, Health motivation; IWD, Intention to Use Wearable Medical Device; UWD, Usage of Wearable Medical Device; CA, Cronbach's Alpha; rho_A, Dijkstra-Hensele's rho; CR, Composite Reliability; AVE, Average Variance Extracted; VIF, Variance Inflation Factors*.

*Author's data analysis*.

**Figure 1:**
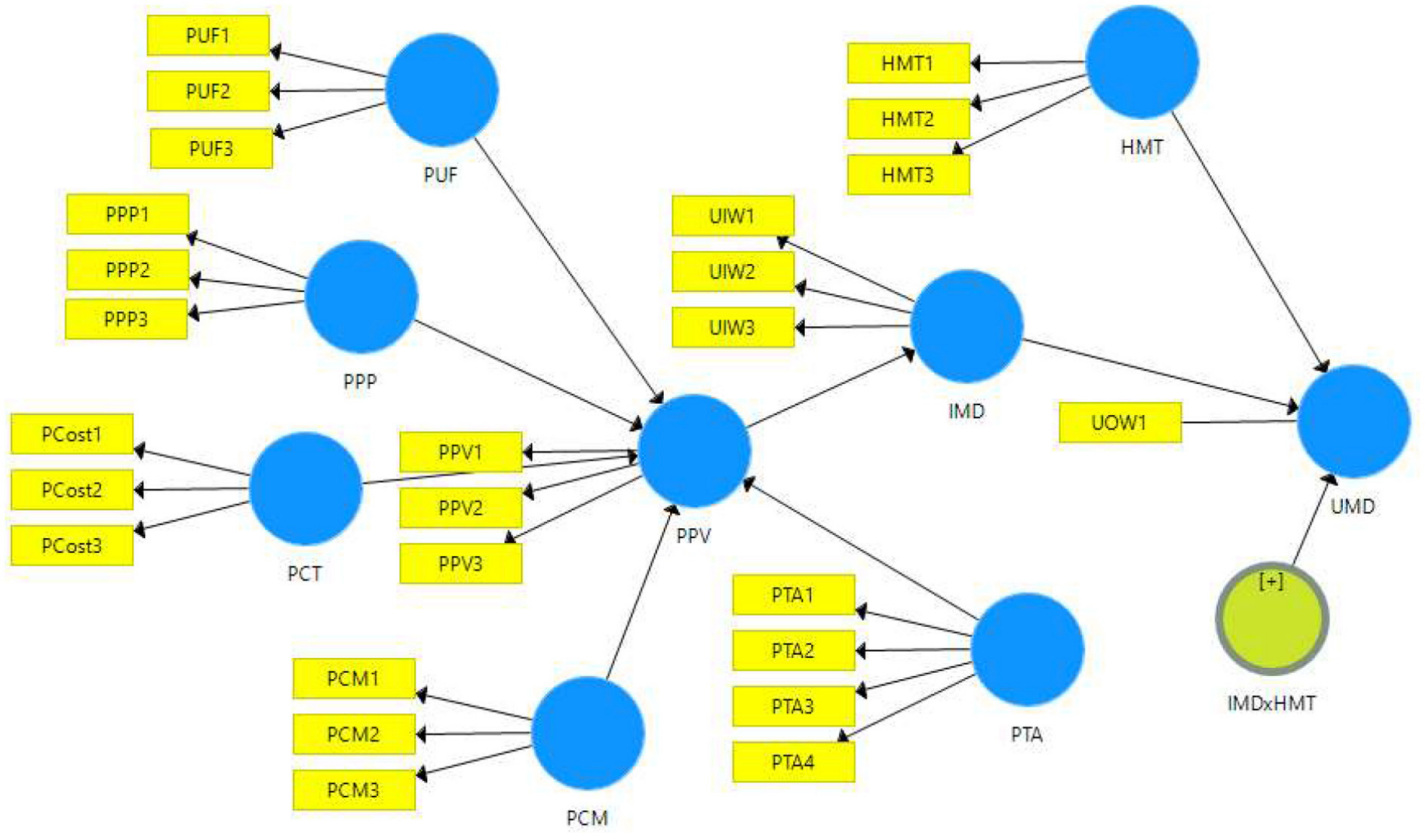
Study model.

**Figure 2:**
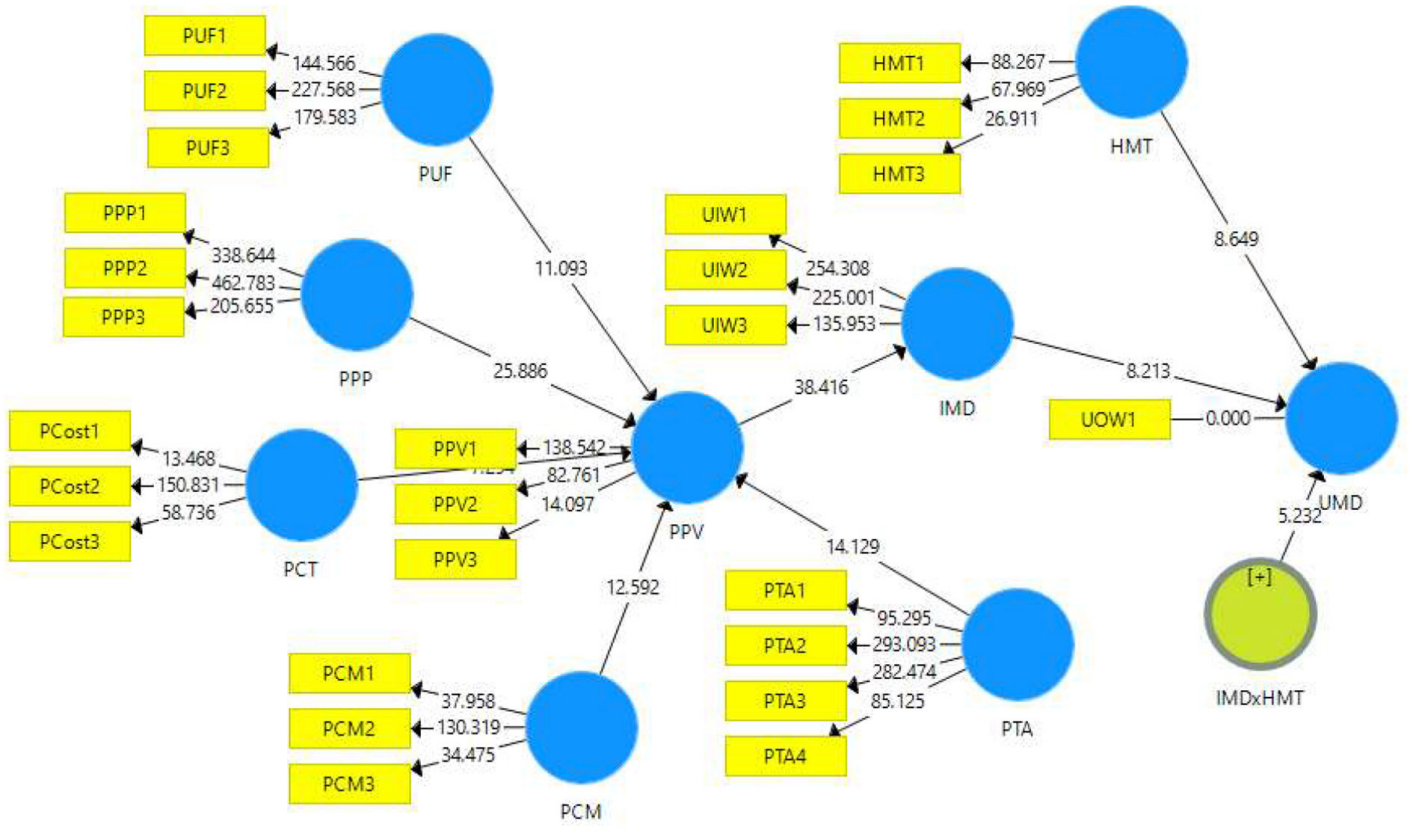
Study model with PLS-SEM results.

Three tests were performed to evaluate the discriminant validity, i.e., the Fornell-Larcker criterion, heterotrait-monotrait (HTMT) ratio, and loading and cross-loading. The Fornell-Larcker criterion was determined using the square root of a particular construct's AVE. The square root of the AVE for every construct must be above the correlation between all the other constructs (38). The results confirmed that discriminant validity was established for the current model (see Table 3). On the other hand, the HTMT ratio values for the study's constructs had acceptable scores, indicating adequate convergent validity (40) (Table 3). Finally, the item loading and cross-loading reported appropriate discriminant validity for the study's constructs (see Appendix 1). Figures 1, 2 (with Findings) depict all hypothesised associations.

**Table 3:** Discriminant validities.

	**PCM**	**PCT**	**PPP**	**PTA**	**PUF**	**PPV**	**HMT**	**IWD**	**UWD**
**Fornell-Larcker criterion**									
PCM	0.886								
PCT	0.518	0.831							
PPP	0.142	0.312	0.900						
PTA	0.274	−0.187	−0.296	0.899					
PUF	0.614	0.047	−0.404	0.852	0.900				
PPV	0.540	0.445	−0.392	0.275	0.490	0.661			
HMT	0.630	0.612	−0.108	−0.246	0.490	0.417	0.584		
IWD	0.796	0.102	−0.128	0.643	0.821	0.615	0.821	0.966	
UWD	−0.489	0.021	0.019	−0.120	−0.353	−0.078	−0.321	−0.416	1.000
**HTMT ratio**									
PCM	-								
PCT	0.681	-							
PPP	0.395	0.548	-						
PTA	0.227	0.391	0.303	-					
PUF	0.649	0.326	0.372	0.864	-				
PPV	0.569	0.515	0.447	0.272	0.520	-			
HMT	0.561	0.680	0.275	0.241	0.227	0.874	-		
IWD	0.788	0.260	0.120	0.630	0.860	0.622	0.154	-	
UWD	0.517	0.113	0.053	0.111	0.378	0.210	0.249	0.423	-

*PCM, Perceived compatibility; PCT, Perceived cost; PPP, Perceived privacy protection; PTA, Perceived technology accuracy; PUF, Perceived usefulness; PPV, Perceived product value; HMT, Health motivation; IWD, Intention to Use Wearable Medical Device; UWD, Usage of Wearable Medical Device*.

***Source:** Author's data analysis*.

#### Study path testing

The adjusted *r*^2^-value for PPV from the five exogenous constructs (i.e., PCM, PCT, PPP, PTA, and PUF) signified that the five factors explained 70.6% of the variation of PPV. Meanwhile, the *Q*^2^-value for this model fragment was 0.468%, which showed high predictive relevance (39).

The path value between PCM and PPV (β = 0.922, *p* = 0.000) revealed that PCM had a positive and significant effect on PPV, thus, supporting H1a. On the other hand, the *f*^2^-value of 0.455 indicated the large effect of PCN on PPV. The path score between PCT and PPV (β = 0.461, *p* = 0.000) showed that PCT had a positive and significant effect on PPV; hence, accepting the H1b. Next, the path coefficients for PPP and PPV (β = −0.893, *p* = 0.000) suggested that the path was negative and significant; therefore, it did not offer support to accept H1c. For PTA and PPV (β = 0.854, *p* = 0.000), a positive and significant effect was exerted on PPV, supporting H1d. An *f*^2^-value of 0.253 indicated the medium effect of PTA on PPV. This outcome confirmed the acceptance of H1d. Lastly, for the path between PUF and PPV (β = −1.186, *p* = 0.000), a negative and significant effect of PUF on PPV was noted, thus, offering no support to accept H1e. All the results are provided in Table 4.

**Table 4:** Path coefficients.

**No**.	**Path**	**Coefficients**	**CI—Min**	**CI—Max**	***T***	***p***	***r^2^***	***Q^2^***	***f^2^***	**Decision**
**Factors affecting the perceived product value of WMDs**										
H_1a_	PCM → PPV	0.922	0.261	0.361	12.741	<0.001	0.706	0.468	0.455	Accepted
H_1b_	PCT → PPV	0.461	0.357	0.561	7.387	<0.001			0.432	Accepted
H_1c_	PPP → PPV	−0.893	−0.957	−0.844	25.926	<0.001			0.946	Rejected
H_1d_	PTA → PPV	0.854	0.771	0.966	14.307	<0.001			0.253	Accepted
H_1e_	PUF → PPV	−1.186	−1.377	−1.034	11.371	<0.001			0.232	Rejected
**Intention to adopt WMDs**										
H_2_	PPV → IWD	0.615	0.589	0.642	38.006	<0.001	0.378	0.347	0.609	Accepted
**Adoption of WMDs**										
H_3_	HMT → UWD	−0.291	−0.348	−0.235	8.531	<0.001	0.261	0.251	0.106	Rejected
H_4_	IWD → UWD	−0.290	−0.345	−0.231	8.351	<0.001			0.088	Rejected

*PCM, Perceived compatibility; PCT, Perceived cost; PPP, Perceived privacy protection; PTA, Perceived technology accuracy; PUF, Perceived usefulness; PPV, Perceived product value; HMT, Health motivation; IWD, Intention to Use Wearable Medical Device; UWD, Usage of Wearable Medical Device*.

***Source:** Author's data analysis*.

The adjusted *r*^2^-value for IWD from the exogenous construct PPV showed that PPV explained 37.8% of the variation of IWD. The *Q*^2^-value for this part of the model was 0.347, showing medium predictive relevance (39). Meanwhile, the path coefficient of the relationship between PPV and IWD (β = 0.615, *p* = 0.000) revealed that PPV had a positive and significant effect on IWD, providing support to accept H2. These results are presented in Table 4.

On the other hand, the adjusted *r*^2^-value for UWD with the two input constructs (i.e., HMT and IWD) demonstrated that HMT and IWD clarified 26.1% of the change in UWD. The *Q*^2^-value of the model was 0.251, showing a medium predictive relevance (39). The effect of HMT on UWD (β =-0.291, *p* = 0.000) suggested that HMT significantly but negatively influenced the use of WMDs and thus, rejected H3. Lastly, IWD (β = −0.290, *p* = 0.000) displayed a negative but significant influence on UWD, providing no evidence to support H4. The results are demonstrated in Table 4.

#### Moderation analysis

**Figure 3:**
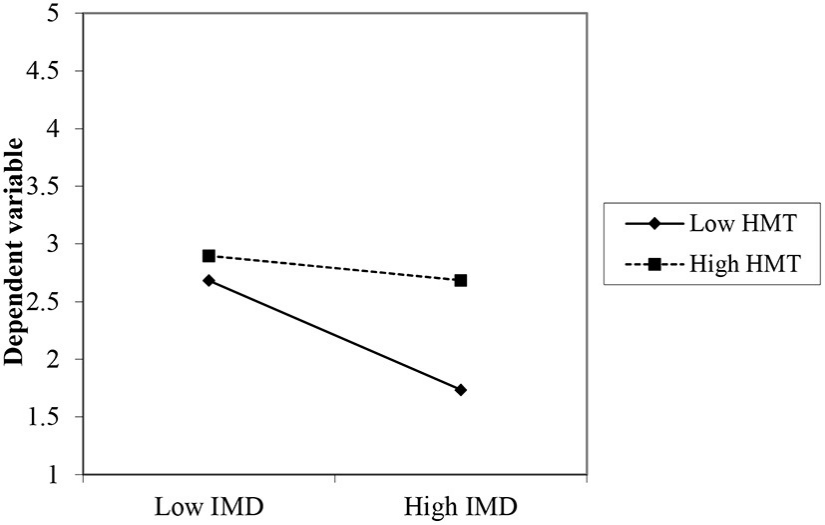
Moderation of HMT between IMD and UWD (Slope Plot).

The moderation analysis revealed that the relationship between IWD and UWD was significantly positively moderated by HMT (β = 0.184, *p* = 0.000), thus, offering substantial evidence to accept HM1. The result is provided in Table 5. Furthermore, the moderation effect is also presented in the slope effect diagram that clearly depicts the significant moderation effect of HMT between the IWD and UWD (see Figure 3). The two lines represent the higher and lower UWD with the HMT. The low level of HMT is one standard deviation unit below the average, and the higher level of HMT is one standard deviation unit above its average. The slope test pieces of evidence that the higher HMT strongly affects the UWD. The lower HMT leads to a lower level of UWD than the higher HMT.

**Table 5:** Moderating effects.

**Hyp**.	**Path**	**Coefficients**	**CI—Min**	**CI—Max**	***t***	***p***	***f*** **^2^**	**Decision**
HMT	IWDxHMT → UWD	0.184	0.122	0.239	5.235	0.000	0.034	Moderation

*PCM, Perceived compatibility; PCT, Perceived cost; PPP, Perceived privacy protection; PTA, Perceived technology accuracy; PUF, Perceived usefulness; PPV, Perceived product value; HMT, Health motivation; IWD, Intention to Use Wearable Medical Device; UWD, Usage of Wearable Medical Device*.

***Source:** Author's data analysis*.

## Discussion and conclusion

This empirical study aimed to determine the critical factors that influenced the intention to use and the adoption of WMDs and the moderating effect of HMT on the relationship between intention to use and adoption of WMDs. The study's findings showed that PCM and PTA positively and significantly impacted PPV. This outcome concurs with the result documented by Yen et al. (21) that technology compatibility influences the perception of technology value and the positive attitude of consumers towards technology.

Next, the result established that PCT significantly influenced the PPV of WMDs. This finding coincides with Sergueeva et al. (7), whereby the perception of technology cost nurtures the technology value and product acceptability to use for personal health screening. The cost of technology shapes the users' benefits and value perception associated with using healthcare technologies (9).

The study outcome also confirmed that the PPP of WMDs significantly but negatively impacted the PPV of WMDs. This result agrees with the finding of Huarng et al. (3) that the perception of personal privacy is negatively associated with the value of healthcare technologies. Wearable technologies involve giving personal information to technology firms, and this reduces the understanding of the WMDs' value (18). Therefore, technology firms need to address privacy issues and improve consumer product value.

Next, PTA significantly influenced the PPV of WMDs. This outcome agrees with the result of Cheung et al. (26), whereby technical accuracy facilitates the perception of technology value. Users believe that reliable and accurate technology offers benefits and raises confidence in the technology (15). The healthcare technology accuracy influences the potential use of the technology to facilitate users in making informed decisions about their health.

The current study's outcome showed that PUF significantly but negatively affected the PPV of WMDs. This result disagrees with Asadi et al. (12), whereby the perceived usefulness has not made users more inclined toward the product value. Malaysian users cannot perceive the WMDs as helpful and do not find it easy to use WMDs, i.e., they find it challenging to utilise the WMDs, lowering the PPV.

The study also found that PPV significantly influenced the intention to use WMDs. This study's result matches the one Yu and Lee (29) reported that perceived value has a positive and significant influence on both attitude and intention to purchase. The perception of benefits and value builds product value and promotes the intention to use healthcare technology (16).

Furthermore, HMT negatively but significantly influenced the usage of WMDs, which did not agree with the outcome in Lee and Lee (1). However, the results support the findings of Yang et al. (23) in the Chinese population, which also showed no positive effect of HMT on the intention to use eDoctor. Malaysian users do not have high HMT, which does not promote the use of healthcare technology. IWD also had a negative but significant impact on WMD usage, which disagreed with the result of Asadi et al. (12). The intention to use is low and does not influence the use behaviour of healthcare technology.

Finally, the moderating analysis confirmed that HMT positively moderated the relationship between the intention to use and the adoption of WMDs. Personal HMT helps to transform intention into usage behaviour.

### Theoretical implications

The current work enhances the theoretical stance that the perception of technology value plays a significant role in influencing the intention to use and the adoption of healthcare technology. The technology attributes offer the perception of technology value as assessing the benefits derived from the WMDs and instigating the intention to use WMDs. Second, the technology attributes help assess the technology value; however, not all the technology attributes can positively influence the technology value. For example, attributes of price and privacy negatively influence the perception of technology value. Lastly, HMT significantly moderates the relationship between the intention to use and the adoption of WMDs. The current study offers evidence that the product value theory is prevalent and significantly explains the intention to use WMDs.

### Practical and managerial implications

This study's findings offer empirical, practical implications for the digital healthcare industry. Personal healthcare technology facilitates the users to manage their personal health and reduce the burden on the public healthcare system. The Malaysian healthcare industry management must understand that Malaysian consumers are more inclined to use WMDs to offer value for WMD consumers. The seller and manufacturers must concentrate on enhancing the usability, and prices can build the users' value (9). This will help to enhance the value perception of the WMDs and promote the intention to use and usage behaviour (41). WMDs' useableness can be boosted by photographic directions simplifying the learning and improving the understanding of the WMDs' features along with the technology value to promote the intention to use WMDs (31).

Furthermore, emergent personal disposition towards health significantly moderates the relationship between the intention to use and the adoption of WMDs. Promoting HMT can help reduce the burden on the public healthcare system (1) and offer individuals a better understanding of health. HMT can be enhanced with health awareness, besides offering incentives to use personal healthcare technology. A better personal HMT can facilitate government spending and reduce the burden on the public healthcare system.

The rise of the wearable medical device industry is closely related to the development of telemedicine. However, as a new industry, wearable medical devices are still in the research and innovation stage of many technologies at this stage. This study provides an essential Practical and Managerial basis for the practical promotion of wearable medical devices. The Practical and Managerial basis of this study is that with the gradual accumulation of research, development and technology, and boosted by increasing application demand, technology developers and software providers can further promote the use of wearable medical devices.

### Study limitations

The current study offers empirical contributions to the literature and for practical purposes. Nonetheless, this study has three limitations. First, the current work assumed a quantitative research design with limited generalisation and did not fully explore the research phenomenon. Future research needs to use fsQCA analysis mixed methods or qualitative research designs to identify further why factors such as health motivation have a negative or no impact on the intention of new medical technologies such as WMD to further promote the adoption of new technologies in the future. Second, this study utilised a limited set of variables influencing the perceived product value. Future investigations must include multiple technological factors (such as the ease of use, support system, social norms, mass adoption, and facilitating conditions) that affect the perceived product value. Lastly, technology adoption is a process that takes time to occur, and adopting a technology may involve the continuous intention to adopt or discard the adoption.

## Data availability statement

The raw data supporting the conclusions of this article will be made available by the authors, without undue reservation.

## Ethics statement

Research Committee in UMK approved the data collection for the current study. The patients/participants provided their written informed consent to participate in this study.

## Author contributions

AS, MM, QY, and NZ: conceptualization, methodology, instrument, and writing—original draft. NH and AA: conceptualization, data collection, formal analysis, and writing—revision. All authors contributed to the article and approved the submitted version.

## Conflict of interest

The authors declare that the research was conducted in the absence of any commercial or financial relationships that could be construed as a potential conflict of interest.

## Publisher's note

All claims expressed in this article are solely those of the authors and do not necessarily represent those of their affiliated organizations, or those of the publisher, the editors and the reviewers. Any product that may be evaluated in this article, or claim that may be made by its manufacturer, is not guaranteed or endorsed by the publisher.

## Appendix

**Appendix 1.** Loading and cross-loading.

	**PCM**	**PCT**	**PPP**	**PTA**	**PUF**	**PPV**	**HMT**	**IWD**	**UWD**
PCM1	*0.846*	0.590	0.501	0.203	0.437	0.303	0.443	0.619	−0.363
PCM2	*0.954*	0.434	−0.095	0.287	0.638	0.651	0.648	0.805	−0.477
PCM3	*0.853*	0.389	0.403	0.170	0.451	0.122	0.511	0.568	−0.486
PCT1	0.526	*0.805*	0.602	−0.291	−0.144	0.110	0.305	0.191	−0.139
PCT2	0.586	*0.932*	0.224	0.098	0.312	0.472	0.574	0.249	−0.012
PCT3	0.268	*0.877*	0.181	−0.461	−0.249	0.371	0.561	−0.143	0.104
PPP1	0.199	0.304	*0.978*	−0.281	−0.337	−0.387	−0.044	−0.064	−0.048
PPP2	0.050	0.288	*0.986*	−0.373	−0.493	−0.447	−0.145	−0.229	0.063
PPP3	0.198	0.336	*0.962*	−0.161	−0.314	−0.270	−0.130	−0.040	0.041
PTA1	0.114	−0.191	−0.373	*0.912*	0.707	0.150	−0.255	0.408	−0.023
PTA2	0.386	−0.166	−0.222	*0.948*	0.678	0.348	−0.169	0.750	−0.205
PTA3	0.300	−0.190	−0.318	*0.979*	0.784	0.240	−0.196	0.636	−0.162
PTA4	0.113	−0.171	−0.268	*0.938*	0.692	0.218	−0.361	0.496	0.022
PUF1	0.671	0.171	−0.195	0.676	*0.933*	0.411	0.208	0.783	−0.352
PUF2	0.452	−0.124	−0.582	0.485	*0.941*	0.569	0.090	0.782	−0.275
PUF3	0.673	0.173	−0.260	0.575	*0.943*	0.335	0.294	0.737	−0.403
PPV1	0.710	0.378	−0.346	0.355	0.654	*0.862*	0.525	0.753	−0.263
PPV2	0.416	0.452	−0.229	0.194	0.354	*0.916*	0.257	0.493	0.019
PPV3	0.031	0.259	−0.440	0.052	0.043	*0.699*	0.157	0.113	0.183
HMT1	0.625	0.548	0.106	−0.363	0.049	0.274	*0.954*	0.143	−0.352
HMT2	0.583	0.601	−0.342	−0.069	0.358	0.526	*0.942*	0.226	−0.276
HMT3	0.372	0.671	−0.288	−0.195	0.094	0.523	*0.832*	−0.022	−0.037
IWD1	0.786	0.058	−0.085	0.511	0.711	0.610	0.200	*0.969*	−0.431
IWD2	0.790	0.197	−0.114	0.691	0.868	0.638	0.202	*0.967*	−0.386
IWD3	0.726	0.032	−0.180	0.669	0.802	0.527	0.107	*0.962*	−0.387
UWD	−0.489	0.021	0.019	−0.120	−0.353	−0.078	−0.321	−0.416	1.000

*PCM, Perceived compatibility; PCT, Perceived cost; PPP, Perceived privacy protection; PTA, Perceived technology accuracy; PUF, Perceived usefulness; PPV, Perceived product value; HMT, Health motivation; IWD, Intention to Use Wearable Medical Device; UWD, Usage of Wearable Medical Device. Italic values are item loadings, and other values are cross-loadings*.

***Source:** Author's data analysis*.
